# Blood Eosinophil Counts in Clinical Trials for Chronic Obstructive Pulmonary Disease

**DOI:** 10.1164/rccm.201912-2384PP

**Published:** 2020-09-01

**Authors:** Dave Singh, Mona Bafadhel, Christopher E. Brightling, Frank C. Sciurba, Jeffrey L. Curtis, Fernando J. Martinez, Cara B. Pasquale, Debora D. Merrill, Norbert Metzdorf, Stefano Petruzzelli, Ruth Tal-Singer, Christopher Compton, Stephen Rennard

**Affiliations:** ^1^ Division of Infection, Immunity, and Respiratory Medicine, University of Manchester, Manchester University National Health Service Hospital Trust, Manchester, United Kingdom; ^2^ Respiratory Medicine Unit, Nuffield Department of Medicine, University of Oxford, Oxford, United Kingdom; ^3^ Institute for Lung Health, National Institute for Health Research Leicester Biomedical Research Centre, Department of Respiratory and Infection Sciences, University of Leicester, Leicester, United Kingdom; ^4^ Division of Pulmonary, Allergy, and Critical Care Medicine, Department of Medicine, University of Pittsburgh School of Medicine, Pittsburgh, Pennsylvania; ^5^ Pulmonary and Critical Care Medicine, VA Ann Arbor Healthcare System, Ann Arbor, Michigan; ^6^ Division of Pulmonary and Critical Care Medicine, University of Michigan, Ann Arbor, Michigan; ^7^ Pulmonary and Critical Care Medicine, Weill Cornell Medical College, New York, New York; ^8^ COPD Patient-Powered Research Network, COPD Foundation, Washington, DC; ^9^ COPD Biomarkers Qualification Consortium, COPD Foundation, Miami, Florida; ^10^ Boehringer Ingelheim International GmbH, Ingelheim am Rhein, Germany; ^11^ Global Clinical Development, Chiesi Farmaceutici, Parma, Italy; ^12^ Global Medical Affairs, Speciality and Primary Care, GlaxoSmithKline, Middlesex, United Kingdom; ^13^ Biopharmaceuticals R&D, AstraZeneca, Cambridge, United Kingdom; ^14^ University of Nebraska Medical Center, Omaha, Nebraska; and; ^15^ Research and Development, AstraZeneca, Gaithersburg, Maryland

Chronic obstructive pulmonary disease (COPD) is a complex condition with pathophysiology and clinical characteristics that vary in presence and severity between patients (1). This variability contributes to the range of treatment responses observed for patients with COPD for both established and experimental therapeutic interventions. Precision medicine is emerging as an approach to combine individual patient clinical characteristics with additional biological information to distinguish among patients with similar diagnoses, with the aim of predicting disease course and treatment response (2). Biomarkers, defined by the U.S. Food and Drug Administration (FDA) as “a defined characteristic that is measured as an indicator of normal biologic processes, pathogenic processes, or responses to an exposure or intervention, including therapeutic interventions” (3), have an important role in precision medicine. Biomarkers offer the ability to enrich clinical trial populations, with the potential to reduce both the cost of drug development and trial failures. Accordingly, the FDA and European Medicines Agency have developed guidance documents to support the qualification of drug development tools, including those for clinical outcome assessments and biomarkers (4, 5).

Increasing awareness of the heterogeneous nature of COPD has led to the concept of “treatable traits.” Treatable traits are disease components that can be individually targeted for treatment (6). Although some treatable traits are identifiable by clinical assessment (e.g., exacerbations), others require investigations, such as imaging for emphysema or detecting biomarkers, to identify the components and/or activity of disease processes (7). Clinical practice and clinical trials are both moving toward the use of biomarkers to improve management and treatment outcomes.

In July 2015, the FDA qualified elevated plasma fibrinogen concentration as the first COPD prognostic or enrichment biomarker for all-cause COPD mortality and COPD exacerbations (8). Elevated fibrinogen has the capacity to improve clinical trial efficiency by facilitating the enrollment of patients who are more likely to experience important clinical outcomes of COPD (e.g., exacerbations). For patients with COPD, blood eosinophil counts (BECs) have the ability to act as a biomarker to identify patients likely to respond to certain treatments (9). Distinguishing among uses of these biomarkers is important. Fibrinogen is a prognostic biomarker, in contrast to BEC, which predicts treatment response. Here, we will discuss the evidence to support the use of BEC as a valuable biomarker in COPD clinical trials.

## Role of Eosinophils in COPD

Eosinophils are granulocytic leukocytes derived from progenitor stem cells in the bone marrow. Their differentiation is stimulated by GM-CSF (granulocyte–monocyte colony–stimulating factor), IL-3 (early phases), and IL-5 (later phases) (10, 11). IL-5 also promotes eosinophil proliferation, trafficking, survival, and degranulation (11). The infiltration of eosinophils into lung tissue is facilitated by locally produced IL-4, IL-13, and CC chemokines (12). Degranulation releases eosinophil-specific basic proteins that are toxic to bronchial epithelial cells (12). T-helper cell type 2 (Th2) inflammation mediators, including IL-5 and eotaxin-2, which have key roles in eosinophil migration, survival, and lung tissue recruitment, are found at greater concentrations in many patients with COPD (13, 14).

Of patients with stable COPD, up to 40% have airway eosinophilia, defined as greater than normal sputum concentration (in studies using normal thresholds of ≥1.1%, >3%, or >3.9% or compared with healthy control subjects) (12, 15–18). There is also evidence that a subgroup of patients with COPD has increased eosinophil numbers in BAL and lung tissue (14, 16). Patients with COPD with higher blood and lung eosinophil numbers have other pathophysiological differences in their lungs, such as greater reticular basement membrane thickening (14). Interestingly, the presence of greater sputum eosinophil counts has been associated with less bacterial colonization in the stable state (19, 20). Of note, the lower respiratory tract microbiome of patients with lower BECs, as assessed by sputum, may have fewer Proteobacteria and an altered Proteobacteria:Firmicutes ratio (21). A recent study reported that low BECs (<100 cells/μl) were associated with increased risks of chronic bacterial infection and pneumonia (22). These findings add to the emerging concept that eosinophil counts and bacterial infection have an inverse relationship in COPD. The mechanism to explain this remains unclear at present, as monoclonal antibodies that lower BECs do not appear to increase the risk of pneumonia (23, 24).

There is also evidence that BECs are increased in patients with COPD compared with age-matched control subjects, even when asthma and atopy are excluded (25). Many studies have reported a relationship between blood and lung eosinophil counts (26–30), suggesting that BEC can be used as a biomarker that reflects the degree of eosinophilic lung inflammation.

Eosinophils are elevated in the airways and blood of a subgroup of patients with COPD during exacerbations (31). Exacerbations associated with elevated eosinophils are related to Th2 inflammation and independent from bacteria- and virus-related exacerbations (31) and account for approximately 30% of all COPD exacerbations (32). Furthermore, patients with COPD with persistently higher BEC at stable state are more likely to experience exacerbations associated with increased sputum eosinophils (33).

The evidence for increased lung eosinophil numbers during the stable state and exacerbations in a subset of patients with COPD suggests that these individuals might benefit from targeted pharmacological treatment directed toward eosinophils themselves and/or toward associated inflammation present in these individuals. As there is a relationship between blood and lung eosinophil counts (14, 26–30), it appears that BECs have potential to act as a biomarker for eosinophil-associated inflammation in the lungs.

## Blood Eosinophils: A Predictive Biomarker of Treatment Response

### Inhaled Corticosteroids

**Figure 1.**
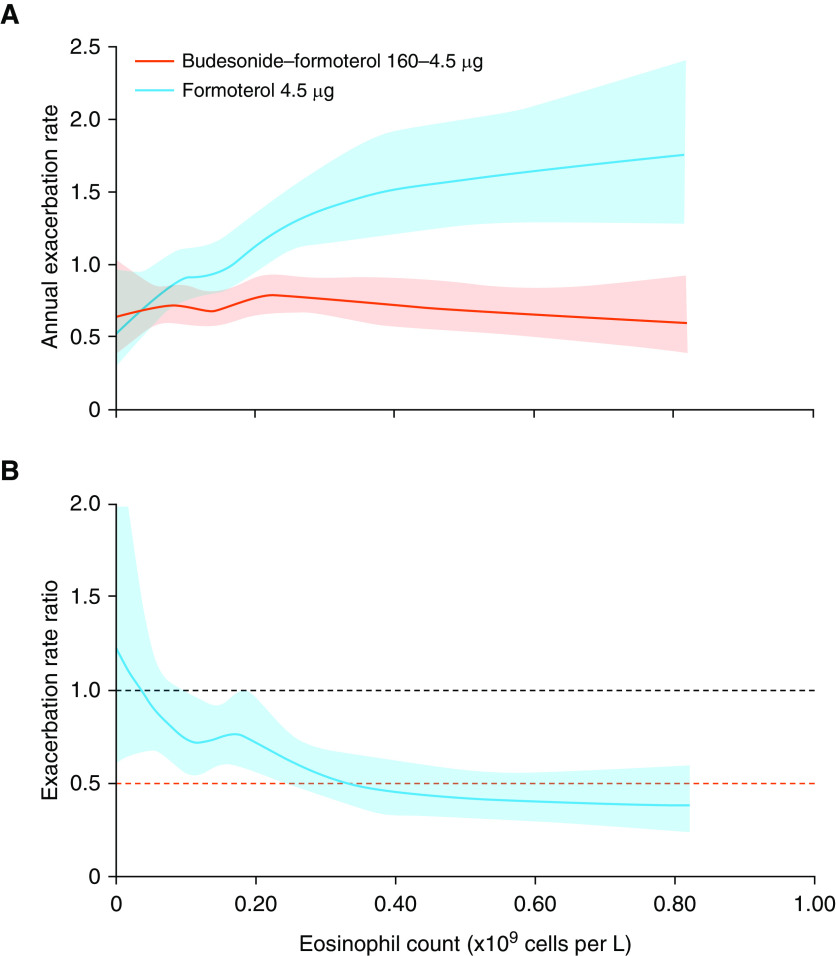
*Post hoc* analysis of (*A*) annual exacerbation rate and (*B*) exacerbation rate reduction by baseline blood eosinophil counts for patients with chronic obstructive pulmonary disease treated with budesonide–formoterol or formoterol. Shaded areas represent 95% confidence interval. Budesonide–formoterol 160–4.5 μg was administered by pressurized metered-dose inhaler (two inhalations). Formoterol 4.5 μg was administered by dry powder inhaler (two inhalations). Reprinted by permission from Reference 36.

Randomized controlled trials (RCTs) have found that patients with COPD with higher sputum eosinophil counts exhibit an increased lung function response to corticosteroids (15, 17, 18). *Post hoc* analyses of RCTs comparing inhaled corticosteroid (ICS)/long-acting β_2_-agonist (LABA) therapy versus LABA monotherapy for patients with COPD with a history of exacerbations have explored the potential for baseline BEC to predict ICS response. These studies demonstrated that the effect of ICSs on exacerbation prevention was larger for patients with higher baseline BECs (Table 1) (34–36). Furthermore, data modeling of RCT data (study numbers: *N* = 1,184; *N* = 3,177 [pooled data]; and *N* = 4,528 [the INCONTROL (Inflammation Control of the Obstructive Lung) analysis; pooled data]; Figure 1) indicated that a beneficial ICS effect occurred at approximately BEC ≥ 100 cells/μl, with a BEC–ICS response relationship observed above this threshold; increasingly greater effects were observed with higher BECs (34–36). These reports highlight that using BECs to predict treatment response in a binomial manner (i.e., responders and nonresponders) does not reflect the complexity of information provided by this biomarker, which can potentially predict different magnitudes of ICS response.

**Table 1.** Summary of Studies Evaluating Eosinophils as a Biomarker to Predict Treatment Response for Patients with Chronic Obstructive Pulmonary Disease

Study	*N*	Key Inclusion Criteria: FEV_1_ and Exacerbation History in Previous Year	Comparison	Results: Treatment Difference for Annual Exacerbation Rate for (a) Overall Population and (b) Blood Eosinophil Analysis
**Studies of ICS/LABA vs. LABA**				
Pascoe *et al.*, 2015 (34)	3,177	FEV_1_ ≤ 70% predicted; ≥1 exacerbation in the previous year	Fluticasone furoate (ICS)/vilanterol (LABA) vs. vilanterol	a. 30% reduction* (44)
				b. BEC ≥ 2%: 29% reduction*; BEC < 2%: 10% reduction*
				BEC ≥2 to <4%: 24% reduction*; BEC 4 to <6%: 32% reduction*; BEC ≥ 6%: 42% reduction*
Siddiqui *et al.*, 2015 (FORWARD) (35)	1,184	FEV_1_ < 50% predicted; ≥1 exacerbation	Beclomethasone dipropionate (ICS)/formoterol (LABA) vs. formoterol	a. 28% reduction
				b. BEC ≥ 279.8 cells/μl: 46% reduction
				BEC < 279.8 cells/μl: 28% reduction
Bafadhel *et al.*, 2018 (36)	4,528	Prebronchodilator FEV_1_ ≤ 50% predicted; ≥1 exacerbation in the previous year	Budesonide (ICS)/formoterol (LABA) vs. formoterol	a. AER: 0.74 vs. 0.79*
				b. BEC < 100 cells/μl: 25% increase to 22% reduction*
				BEC 100–190 cells/μl: 25% reduction*
				BEC 200–340 cells/μl: 26–50% reduction*
				BEC 350–630 cells/μl: 51–60% reduction*
**Studies of ICS/LABA vs. LABA/LAMA**				
Lipson *et al.*, 2018 (IMPACT) (37)	6,204	FEV_1_ < 50% predicted and ≥1 moderate to severe exacerbation OR FEV_1_ 50–80% predicted and ≥2 moderate exacerbations or 1 severe exacerbation	Fluticasone furoate (ICS)/vilanterol (LABA) vs. umeclidinium (LAMA)/vilanterol (LABA)	a. AER: 1.07 vs. 1.21*
				b. BEC ≥ 150 cells/μl: 1.08 vs. 1.39*
				BEC < 150 cells/μl: 1.06 vs. 0.97*
Wedzicha *et al.*, 2016 (FLAME) (47)	3,362	Post-bronchodilator FEV_1_ ≥25% to <60% predicted; ≥1 exacerbation	Glycopyrronium (LAMA)/indacaterol (LABA) vs. fluticasone (ICS)/salmeterol (LABA)	a. 11% reduction^†^ (LAMA/LABA vs. ICS/LABA)
*Post hoc* analysis (Roche *et al.*, 2017) (48)		Patients with BEC > 600 cells/μl were excluded		b. BEC < 2%: 20% reduction^†^ (LAMA/LABA vs. ICS/LABA)
				BEC ≥ 2%: 15% reduction^†^ (LAMA/LABA vs. ICS/LABA)
				a. 17% reduction^†^ (LAMA/LABA vs. ICS/LABA)
				b. BEC < 150 cells/μl: 28% reduction^†^ (LAMA/LABA vs. ICS/LABA)
				BEC 150 to <300 cells/μl: 11% reduction^†^ (LAMA/LABA vs. ICS/LABA)
				BEC 300–600 cells/μl: 7% reduction^†^ (LAMA/LABA vs. ICS/LABA)
**Studies of ICS/LABA/LAMA vs. LABA/LAMA or LAMA**				
Papi *et al.*, 2018 (TRIBUTE) (38)	1,532	FEV_1_ < 50% predicted; ≥1 moderate to severe exacerbation in the previous year; receiving inhaled maintenance medication	Beclomethasone dipropionate (ICS)/formoterol fumarate (LABA)/glycopyrronium (LAMA) vs. indacaterol (LABA)/glycopyrronium (LAMA)	a. 15% reduction*
				b. BEC < 200 cells/μl: 13% reduction*
				BEC ≥ 200 cells/μl: 20% reduction*
Lipson *et al.*, 2018 (IMPACT) (37)	10,355	FEV_1_ < 50% predicted and ≥1 moderate to severe exacerbation OR FEV_1_ 50–80% predicted and ≥2 moderate exacerbations or 1 severe exacerbation in the previous year	Fluticasone furoate (ICS)/vilanterol (LABA)/umeclidinium (LAMA) vs. fluticasone furoate (ICS)/vilanterol (LABA) vs. umeclidinium (LAMA)/vilanterol (LABA)	a. ICS/LABA/LAMA vs. LABA/LAMA: 25% reduction*
				ICS/LABA/LAMA vs. ICS/LABA: 15% reduction*
				b. ICS/LABA/LAMA vs. LABA/LAMA, BEC ≥ 150 cells/μl: 32% reduction*
				ICS/LABA/LAMA vs. LABA/LAMA, BEC < 150 cells/μl: 12% reduction*
Vestbo *et al.*, 2017 (TRINITY) (39)	2,691	FEV_1_ < 50%; ≥1 moderate to severe COPD exacerbation	Beclomethasone dipropionate (ICS)/formoterol fumarate (LABA)/glycopyrronium (LAMA) (fixed triple) vs. tiotropium (LAMA) and beclomethasone dipropionate (ICS)/formoterol fumarate (LABA)/tiotropium (LAMA) (open triple)	a. Fixed 20% reduction, open 21% reduction*
				b. BEC < 2%: fixed 7% reduction,* open 9% reduction*
				BEC ≥ 2%: fixed 30% reduction,* open 31% reduction*
				BEC < 200 cells/μl: fixed 8% reduction,* open 9% reduction*
				BEC ≥ 200 cells/μl: fixed 36% reduction,* open 38% reduction*
**ICS withdrawal studies**				
Watz *et al.*, 2016 (WISDOM) (49)	2,296	FEV_1_ < 50% predicted; ≥1 exacerbation	Tiotropium (LAMA)/salmeterol (LABA)/fluticasone (ICS). Two arms: first group continues treatment for 52 wk; second initiates stepwise reduction of ICS every 6 wk down to placebo	a. ICS withdrawal vs. continuation: 10% increase*
				b. BEC < 2%: 2% increase*; BEC ≥ 2%: 22% increase*; BEC ≥ 4%: 63% increase*; BEC ≥ 5%: 82% increase*
				BEC < 300 cells/μl*: 4% increase*; BEC ≥ 300 cells/μl*: 56% increase*
				BEC < 400 cells/μl: 7% increase*; BEC ≥ 400 cells/μl: 73% increase*
Calverley *et al.*, 2017 (50)	2,420			b. ≥1 exacerbation in prior year AND: BEC ≥ 300 cells/μl: 45% increase*; BEC ≥ 400 cells/μl: 25% increase*≥2 exacerbations in prior year AND: BEC ≥ 300 cells/μl: 75% increase*; BEC ≥ 400 cells/μl: 196% increase*
		
Chapman *et al.*, 2018 (SUNSET) (51)	527	Post-bronchodilator FEV_1_ ≥40% to <80% predicted; ≤1 exacerbation	Tiotropium (LAMA)/salmeterol (LABA)/fluticasone (ICS). Two arms: first group continues with triple therapy; second switches to glycopyrronium (LAMA)/indacaterol (LABA)	a. ICS withdrawal vs. continuation 8% increase*
				b. BEC ≥ 300 cells/μl: 86% increase*
**Studies of monoclonal antibodies**				
Pavord *et al.*, 2017 (METREX) (23)	462	Post-bronchodilator FEV_1_ >20% to ≤80% predicted; ≥2 moderate or ≥1 severe exacerbation; BEC ≥ 150 cells/μl at baseline or ≥300 cells/μl in the previous year	Mepolizumab vs. placebo	a. 2% reduction*
				b. BEC ≥ 150 cells/μl at baseline or ≥300 cells/μl in the prior year: 18% reduction*
Pavord *et al.*, 2017 (METREO) (23)	675	Post-bronchodilator FEV_1_ >20% to ≤80% predicted; ≥2 moderate or ≥1 severe exacerbation; BEC ≥ 150 cells/μl at baseline or ≥300 cells/μl in the previous year	Mepolizumab vs. placebo	a. NA (all patients had BEC ≥ 150 cells/μl at screening or ≥300 cells/μl during the previous year)
				b. BEC ≥ 150 cells/μl at screening or ≥300 cells/μl during the previous year: 20% reduction*
Criner *et al.*, 2019 (GALATHEA) (24)	1,656	Post-bronchodilator FEV_1_ >20% to ≤65% predicted; ≥2 moderate or ≥1 severe exacerbation; BEC ≥ 220 cells/μl	Benralizumab vs. placebo	a. NA
				b. BEC ≥ 220 cells/μl: 30 mg, 4% reduction; 100 mg, 17% reduction*
Criner *et al.*, 2019 (TERRANOVA) (24)	2,254	Post-bronchodilator FEV_1_ >20% to ≤65% predicted; ≥2 moderate or ≥1 severe exacerbation; BEC ≥ 220 cells/μl	Benralizumab vs. placebo	a. NA
				b. BEC ≥ 220 cells/μl: 10 mg, 15% reduction; 30 mg, 4% increase; 100 mg, 7% reduction*
Criner *et al.*, 2019 (GALATHEA/TERRANOVA prespecified analysis of pooled data) (24)	2,665	Post-bronchodilator FEV_1_ >20% to ≤65% predicted; ≥2 moderate or ≥1 severe exacerbation; BEC ≥ 220 cells/μl	Benralizumab (100 mg) vs. placebo	a. NA
				b. BEC ≥ 220 cells/μl: 12% reduction*
				BEC ≥ 220 cells/μl AND: ≥3 exacerbations in prior year: 31% reduction*
				FEV_1_ < 40% predicted: 24% reduction*
				Post-bronchodilator response ≥ 15%: 33%
				≥3 exacerbations in the prior year and receiving triple therapy: 30% reduction*
**Studies of PDE4 inhibitors**				
Martinez *et al.*, 2018 (REACT/RE^2^SPOND) (53)	4,299	FEV_1_ ≤ 50% predicted; ≥2 exacerbations	Roflumilast vs. placebo	a. 12% reduction*
				b. BEC ≥ 150 cells/μl: 19% reduction*
				BEC ≥150 to <300 cells/μl: 16% reduction*
				BEC ≥ 300 cells/μl: 23% reduction*
				Prior hospitalization for COPD exacerbation AND:
				BEC ≥ 150 cells/μl: 35% reduction*
				BEC ≥ 300 cells/μl: 43% reduction*

*Definition of abbreviations*: AER = annualized exacerbation rate; BEC = blood eosinophil count; COPD = chronic obstructive pulmonary disease; FORWARD = Foster 48-Week Trial to Reduce Exacerbations in COPD; ICS = inhaled corticosteroids; IMPACT = Informing the Pathway of COPD Treatment; LABA = long-acting β_2_-agonist; LAMA = long-acting muscarinic antagonist; METREO = Mepolizumab vs. Placebo as Add-on Treatment for Frequently Exacerbating COPD Patients Characterized by Eosinophil Level; METREX = Mepolizumab vs. Placebo as Add-on Treatment for Frequently Exacerbating COPD Patients; NA = not available; PDE4 = phosphodiesterase-4; REACT = Roflumilast in the Prevention of COPD Exacerbations While Taking Appropriate Combination Treatment; RE^2^SPOND = Roflumilast Effect on Exacerbations in Patients on Dual (LABA/ICS) Therapy; SUNSET = Study to Understand the Safety and Efficacy of ICS Withdrawal from Triple Therapy in COPD; WISDOM = Withdrawal of Inhaled Steroids during Optimized Bronchodilator Management.

* Exacerbation rate for moderate and severe exacerbations.

^†^ Exacerbation rate for mild, moderate, and severe exacerbations.

Three studies (IMPACT [Informing the Pathway of COPD Treatment; NCT02164513], TRIBUTE [NCT02579850], and TRINITY [NCT01911364]) used BEC to predict response to triple therapy (ICS/LABA/long-acting muscarinic antagonist [LAMA]) compared with dual therapy (LABA/LAMA) or LAMA monotherapy for patients with COPD with a history of exacerbations (Table 1) (37–39). In IMPACT, there was a substantially greater reduction in annual exacerbation rate (AER) with ICS/LABA/LAMA compared with LABA/LAMA for patients with COPD who had BEC ≥ 150 cells/μl versus <150 cells/μl (32% vs. 12%, respectively) (37). In TRIBUTE, BEC ≥ 2% compared with <2% demonstrated a greater ICS effect on exacerbation prevention (19% vs. 6%, respectively) (38). Results were similar in TRINITY, where BEC ≥ 2% or ≥200 cells/μl demonstrated 30% AER reduction with triple therapy versus LAMA monotherapy compared with reductions of ≤10% below these thresholds (39).

**Figure 2.**
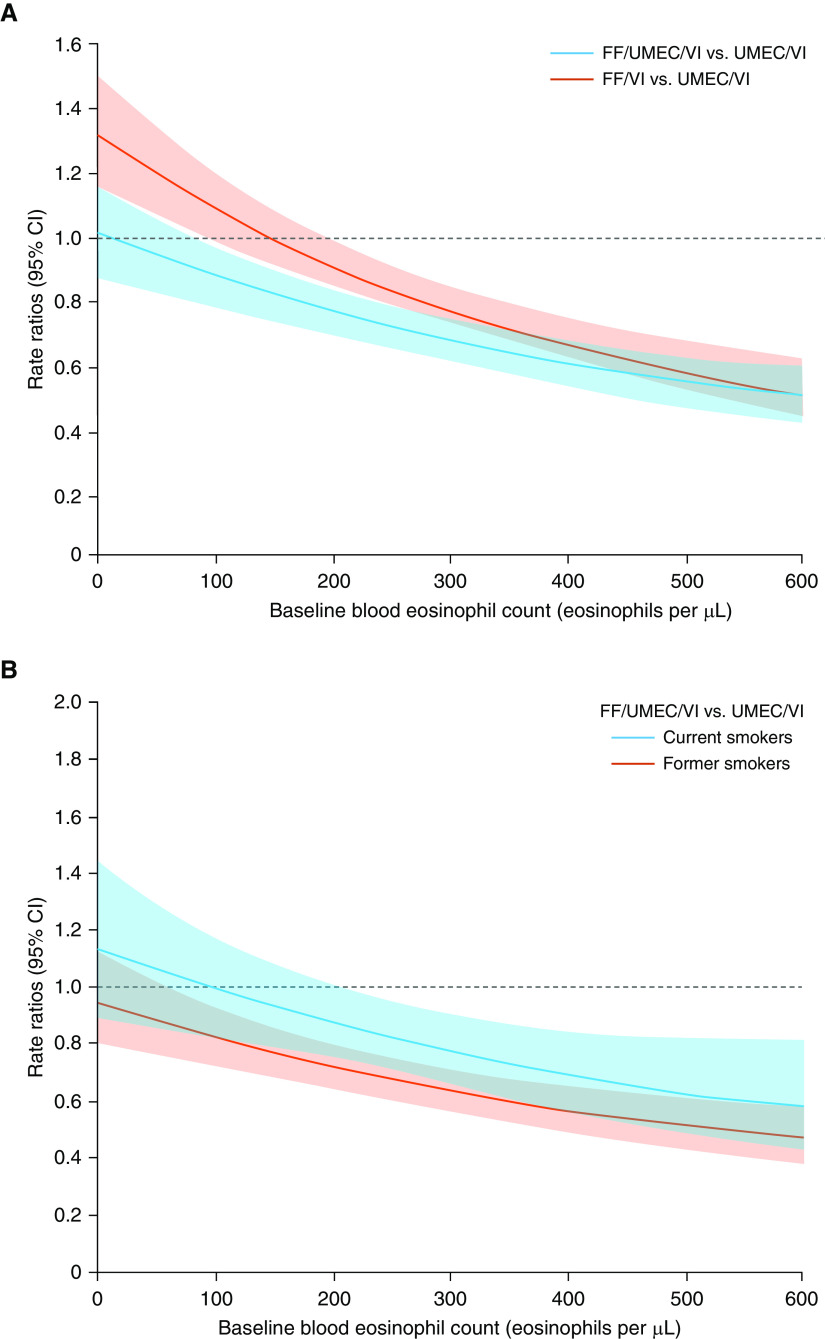
Prespecified analysis of between treatment ratios for rates of moderate or severe exacerbations by baseline blood eosinophil counts for patients with chronic obstructive pulmonary disease treated with (*A*) triple or dual therapy containing inhaled corticosteroids compared with dual therapy, and (*B*) triple therapy containing inhaled corticosteroids compared with dual therapy in smokers and former smokers. CI = confidence interval; FF = fluticasone furoate; UMEC = umeclidinium; VI = vilanterol. Reprinted by permission from Reference 40.

Prespecified modeling of IMPACT data for patients with available baseline BEC data (*N* = 10,333) demonstrated a BEC–ICS response relationship for exacerbation prevention, with ICS benefits apparent at approximately ≥100 cells/μl and greater effects at higher BECs (Figure 2) (40). BEC also predicted treatment effects on lung function and health-related quality of life, although these results were less consistent. Importantly, the ICS effect was reduced for current smokers, thereby increasing the BEC threshold above which ICS benefits were observed for current smokers (Figure 2) (40). A similar pattern with regard to current smoking was observed in the INCONTROL *post hoc* analysis (36). Consistent with the IMPACT analysis, the INCONTROL analysis also indicated that BEC predicted treatment effects on lung function and health-related quality of life (36).

Reduced ICS treatment effect for current smokers was also reported in a *post hoc* analysis of the SUMMIT (Study to Understand Mortality and Morbidity in COPD) (NCT01313676) trial (41) and for patients with asthma (42, 43). Other RCTs have not demonstrated that current smoking reduces ICS effects (44–46), although it should be noted that the larger sample of the pooled analyses described previously (36, 40) increases statistical power. The mechanisms for decreased ICS sensitivity in current smokers have not been clearly defined. These results highlight how clinical characteristics, in this case current smoking, can modify the interpretation of BEC for predicting treatment response.

Treatment response using BEC has also been compared for ICS-containing dual treatment versus non-ICS dual treatment for patients with COPD (Table 1). In IMPACT, ICS/LABA was superior to LABA/LAMA treatment for AER reduction in the overall population (10% mean difference) (37). In contrast, the FLAME (NCT01782326) study found that LAMA/LABA had overall superiority to ICS/LABA for reducing AER (17% mean difference) (47). These increased ICS effects in IMPACT compared with FLAME may be explained by a greater exacerbation risk for the IMPACT study populations, with more patients having two or more moderate exacerbations or one or more severe exacerbation in the previous year (37, 47). There are also differences in the study designs that likely influenced the results; patients took their own inhaled treatments during the 2-week run-in period in IMPACT, whereas in FLAME there was a 4-week run-in period with LAMA monotherapy. ICS withdrawal occurred at randomization in IMPACT (for patients randomized to LAMA/LABA), whereas in FLAME, ICSs were withdrawn before run-in and were reintroduced at randomization (for the ICS/LABA group). Data modeling of IMPACT found a larger effect of ICS/LABA versus LAMA/LABA for exacerbation prevention at higher BEC (40). In contrast, a *post hoc* analysis of FLAME found little difference between treatments at higher BECs (48) (Table 1). This disparity highlights again how different clinical characteristics (i.e., increased exacerbation risk in IMPACT) can alter patients’ sensitivity to ICSs and thereby also change the treatment effect at different BEC thresholds.

*Post hoc* analyses of stepped ICS withdrawal for patients receiving triple inhaled therapy (WISDOM [Withdrawal of Inhaled Steroids during Optimized Bronchodilator Management; NCT00975195] trial) reported that deleterious effects of ICS withdrawal were observed only for patients with BEC ≥ 300 cells/μl, with the greatest effect observed for those who also had a history of two or more exacerbations (Table 1) (49, 50). This result further supports the concept of greater ICS treatment effects for patients with greater exacerbation risk and higher BEC. The SUNSET (Study to Understand the Safety and Efficacy of ICS Withdrawal from Triple Therapy in COPD; NCT02603393) study, which enrolled patients receiving triple therapy who had no more than one exacerbation in the previous year, also found that disease deterioration, including greater exacerbations, was most clearly observed for patients with BEC ≥ 300 cells/μl (Table 1) (51).

### Monoclonal Antibodies

The monoclonal antibodies mepolizumab (anti–IL-5) and benralizumab (anti–IL-5 receptor alpha [IL-5Rα]) have been evaluated for patients with COPD with a history of two or more moderate COPD exacerbations or one or more severe exacerbations in the previous year (Table 1). Mepolizumab significantly reduced AER for patients with BEC ≥ 150 cells/μl at screening or ≥300 cells/μl during the previous year versus placebo in METREO (Mepolizumab vs. Placebo as Add-on Treatment for Frequently Exacerbating COPD Patients Characterized by Eosinophil Level; NCT02105948) but not METREX (Mepolizumab vs. Placebo as Add-on Treatment for Frequently Exacerbating COPD Patients; NCT02105961) (both phase III trials) (23). Interestingly, with consideration of exacerbations only involving oral corticosteroid treatment, the effect of mepolizumab was increased (23). For benralizumab versus placebo in both the GALATHEA (NCT02138916) and TERRANOVA (NCT02155660) phase III trials, reduction in AER did not reach statistical significance for the primary analysis population with BEC ≥ 220 cells/μl (24). At face value, the absence of a conclusive treatment effect in these four clinical trials (total *n* = 5,422) might raise concerns regarding the biological plausibility of eosinophils as a biomarker in COPD. However, prespecified analyses of GALATHEA and TERRANOVA indicated that the combination of BEC ≥ 220 cells/μl, three or more exacerbations in the prior year, and triple inhaled therapy identified patients who experienced the greatest treatment effect with benralizumab for reduction of AER (52). Thus, these results support the continued use of designs that combine clinical characteristics and BECs to identify responder populations among patients with COPD at high risk for frequent exacerbations.

### Phosphodiesterase-4 Inhibitors

A predefined pooled analysis of the phosphodiesterase-4 inhibitor roflumilast trials (REACT [Roflumilast in the Prevention of COPD Exacerbations While Taking Appropriate Combination Treatment; NCT01329029]/RE^2^SPOND [Roflumilast Effect on Exacerbations in Patients on Dual (LABA/ICS) Therapy; NCT01443845]) found that a combination of BEC ≥ 300 cells/μl and one or more prior hospitalization for COPD exacerbation was associated with a 43% reduction in moderate to severe exacerbations for patients receiving roflumilast versus placebo compared with a reduction of 12% for the overall population (Table 1) (53). The ROBERT (Roflumilast Biopsy European Research Trial; NCT01509677) study demonstrated a significant reduction in eosinophils in sputum and bronchial biopsy samples, but not in the blood, with roflumilast treatment, providing evidence for phosphodiesterase-4 inhibition acting through modulation of lung eosinophil numbers (54).

## BEC: Cohort Studies

Cohort studies evaluating BEC as a prognostic COPD biomarker have provided inconsistent results, particularly for the association between BEC and exacerbations (26, 55–57). This inconsistency led to doubts about the utility of BEC as a COPD biomarker (58). Analysis of data from RCTs demonstrated that higher BECs are associated with future exacerbation risk in the non-ICS treatment arms. Many cohort studies have not found this association, for the following reasons: *1*) cohort studies have included patients with no prior exacerbation history, in contrast to the RCTs, which focused only on patients with an exacerbation history; *2*) there was no relationship between BEC and exacerbation rates in the ICS treatment groups of RCTs; and *3*) the inclusion of patients receiving ICS in cohort studies reduces the ability of BECs to predict exacerbation rates. Nevertheless, analysis of larger cohorts indicates that in the subgroup of patients with greater exacerbation risk (two or more exacerbations in the previous year), a higher BEC is associated with increased exacerbation rates during prospective follow-up (59).

## Methodology Issues Regarding Eosinophils as a Biomarker

### Relationship between Lung and BEC

#### Correlation between sputum and BEC

Although sputum induction is a practical method for assessing airway inflammation, it has some limitations. It is unsuitable for point-of-care testing, requires expertise, and is not always successful (up to 30% failure rate) (27, 60, 61). Eosinophil detection is more accessible by blood than sputum. For patients with COPD in the stable state, a statistically significant but moderate correlation exists between BEC and sputum eosinophils (correlation coefficient values range from 0.18–0.54) (27–30), although the SPIROMICS (SubPopulations and InteRmediate Outcome Measures in COPD Study) (NCT01969344) cohort indicated only a weak correlation (26). A BEC of ≥215 cells/μl, >265 cells/μl, or >400 cells/μl had a sensitivity of 60%, 72%, and 71% and specificity of 93%, 56%, and 91%, respectively, for identifying sputum eosinophilia (≥3%) (30, 62). A COPD disease stable state BEC of >300 cells/μl identified patients with sputum eosinophilia (≥3%) in 71% of cases (29).

Assumptions have been made that BEC should correlate strongly with sputum eosinophils to be a relevant biomarker in clinical practice. However, eosinophils are known to migrate to all tissues and largely reside in the gastrointestinal tract (63). Therefore, a perfect correlation is unlikely to exist between sputum and BEC. In the current literature, the utility of BECs as a biomarker in COPD is often dismissed, because only a weak correlation to sputum count was found in the SPIROMICS cohort (26). Limitations of some previous studies include multiple sites being used for sputum and blood processing, which can lead to variability between individual observers for sputum counting and variation in the quality of sputum obtained. Furthermore, rounding of BECs to one significant figure decreases the ability to observe a relationship; for example, in the SPIROMICS (NCT01969344) multicenter cohort, an excessive number of patients had BECs with only one significant figure (26).

#### Correlation between BEC and lung tissue eosinophilia

Studies have demonstrated both an association and no association between BEC and tissue eosinophilia (64, 65). Study results may be affected by the tissue source (endobronchial biopsy vs. lung tissue). Nevertheless, a study in patients with COPD without any previous diagnosis of asthma and who were atopy negative (by skin prick testing) with higher and lower BECs (>250 and <150 cells/μl, respectively) demonstrated significantly more eosinophils in sputum, BAL, and bronchial mucosal tissue in the higher BEC group (14).

### BEC Measurement Methodology

The reproducibility of different methods and equipment to detect BEC has been evaluated. In clinical practice, BECs are routinely measured using FDA-endorsed analyzers. A study comparing different Coulter counters for leukocyte differential cell counts found greater error and reduced reproducibility with the VCS technology than with the Technicon H-1 instrument (66). BEC can be measured using point-of-care tests such as HemoCue WBC DIFF System, which has demonstrated a close correlation (*r* = 0.85) between this method and with standard venipuncture laboratory analysis (Abbott Architect ci8200 analyzer), which was unaffected by presence of asthma or COPD (67). In a repeated-sampling substudy of the HemoCue WBC DIFF System for patients with COPD, the intraclass correlation coefficient (ICC) of total eosinophils was 0.90 (95% confidence interval [CI], 0.73–0.96), with a Cronbach α of >0.95 (67).

Relative eosinophil counts (number of eosinophils per 100 cells) was the standard way of evaluating blood cells until the emergence of modern technology facilitating absolute counts. However, relative counts are more accurate than absolute counts and provide further information on presence of other cell types (68). In contrast, absolute counts are likely to give information about the burden of eosinophils and associated mediators. Absolute counts are, however, affected by the accuracy of the reading (i.e., precision of the estimate) and the method of reporting (68).

### Stability of BEC

The stability of repeated sputum eosinophil counts has a reported ICC of 0.63 and 0.49 over 2 and 12 weeks, respectively (69, 70). Repeated measures analysis of BECs for patients with COPD from over 3 months to 2 years have found that the ICC for BECs ranged from 0.64 to 0.89 (Table 2) (28, 57, 71–75). These ICC values are comparable or greater than other routinely used biomarkers (i.e., cholesterol [ICC, 0.72–0.81] or glycated Hb [ICC, 0.59]) (76–78). These values are also comparable to that for fibrinogen, the FDA-approved prognostic biomarker for COPD (8). For healthy individuals, fibrinogen demonstrated an ICC of 0.79 over 1 year (78).

**Table 2.** Summary of Studies on the Stability of Blood Eosinophil Count Measures for Patients with Chronic Obstructive Pulmonary Disease

Study	*N*	Patient Group	Assessment Period	Type of Analysis	Main Results
					
Long *et al.*, 2020 (72)	255	Patients with stable COPD	1 yr	Spearman’s rank correlation; repeatability (ICC); Bland-Altman regression analysis; repeatability coefficient analysis	Spearman’s rank correlation at 12 mo: 0.71; *P* < 0.001
					ICC at 12 mo: 0.84
					Bland-Altman regression at 12 mo: *P* < 0.001
Southworth *et al.*, 2018 (73)	82	Patients with stable COPD (>4 wk from exacerbation)	6 mo, >2 yr	Spearman’s rank correlation; repeatability (ICC); Bland-Altman regression	Spearman’s rank correlation at 6 mo: 0.80; *P* < 0.001
					ICC at 6 mo: 0.89
					Spearman’s rank correlation at >2 yr: 0.74; *P* < 0.001
					ICC at >2 yr: 0.87
					Bland-Altman regression: 6 mo, *P* = 0.006; >2 yr, *P* = 0.015
Barker, 2012 (74)	145	Patients with stable COPD (over 3–6 mo)	6 mo	Repeatability (ICC)	ICC at 3 mo: 0.66
					ICC at 6 mo: 0.73
Landis, 2017 (75)	27,557	Primary care cohort of patients with COPD with stable disease	1 yr	Repeatability (ICC); sensitivity analysis excluded patients who had been prescribed OCS or antibiotics during follow-up	ICC at 1 yr full cohort: 0.64
					ICC at 1 yr sensitivity cohort: 0.70
Bafadhel *et al.*, 2017 (71)	1,483	Patients receiving ICS	1 yr	Repeated measures analysis of BEC once every 3 mo in a 12-mo minimum period (ICC)	ICC over 3 mo: 0.79; 65% of patients with COPD remained above or below the BEC cutoff of 400 cells/μl during the year
Singh *et al.*, 2014 (28)	1,483	ECLIPSE study cohort	3 yr	Four BEC measurements over 3 yr analyzed through BEC cutoffs	37% of patients had persistent BEC ≥ 2%
Casanova *et al.*, 2017 (57)	CHAIN, 424; BODE, 308	Patients with COPD from the CHAIN study cohort and BODE study cohort	2 yr	Analysis through a cutoff of BEC ≥ 300 cells/μl	Over 2 yr, 16% of patients in the CHAIN cohort and 12% of patients in the BODE cohort had persistent BEC ≥ 300 cells/μl

*Definition of abbreviations*: BEC = blood eosinophil count; BODE = body mass index, degree of airflow obstruction, functional dyspnea, and exercise capacity; CHAIN = COPD History Assessment in Spain; COPD = chronic obstructive pulmonary disease; ECLIPSE = Evaluation of COPD Longitudinally to Identify Predictive Surrogate End-points; ICC = intraclass coefficient; ICS = inhaled corticosteroids; OCS = oral corticosteroids.

Confusion about the stability of repeated BEC measurements over time is partly because studies have often used arbitrary cutoff thresholds, most frequently 2% or ≥150 cells/μl. This approach is limited, because a group of patients close to a chosen threshold may cross the threshold despite experiencing only a small absolute change (72, 73). Such an effect will be worse when multiple repeated measurements are taken; for example, categorizing patients as having BECs above versus below 2% or 150 cells/μl over 3 years (testing yearly) determined that only 51% of patients with COPD remained consistently either above or below these cutoffs (71). It is not intuitive to conclude that BECs are not stable because they fall on either side of an arbitrarily chosen cutoff. Nevertheless, evaluation of long-term repeated BEC measurements over 2 years for patients with COPD with eosinophil counts <150 cells/μl found most (≥86%) measurements remained in the same BEC category during this period (Table 2) (73). Furthermore, evaluation of data from IMPACT found that choosing the mean, median, or the greatest or least BEC (between two results) had no impact on the ability of BECs to predict ICS response for the prevention of exacerbations (79).

## BEC as a Biomarker of Treatment Response: Integration of the Evidence

The data reviewed illustrate that BECs are correlated with lung eosinophil numbers and that stability of repeated measurements over time, when assessed by standard statistical methods such as ICC, is very similar to other biomarkers used in clinical practice. Negative views about the variability of BECs have arisen from studies that concluded that a lack of stability was related to variations across a BEC threshold, but such an approach has limited statistical validity.

RCTs conducted in patients with COPD with a history of exacerbations have consistently produced evidence for a greater effect of ICS at higher BECs. Large data sets (for example, *N* = 4,528 and *N* = 10,333) have demonstrated a continuous BEC–ICS response relationship (36, 40). Although many biomarkers are used to dichotomize a population, BECs require a more sophisticated approach. Different BEC thresholds define subgroups of patients with different magnitudes of ICS response; for example, ≥100 cells/μl defines a large subgroup where ICS response is more likely, whereas higher thresholds define smaller subgroups where larger treatment effects can be expected (9).

The analyses of different drug classes illustrate that integration of clinical characteristics in combination with BECs is needed to define COPD subgroups with increased likelihood of a positive response to a pharmacological intervention. For example, the level of exacerbation risk (i.e., two moderate/one severe vs. one moderate exacerbation in the previous year) and current smoking status influence the ICS response at different BEC values (40, 80). Furthermore, anti–IL-5Rα treatment may have greater utility for a subgroup of patients who are already on triple inhaled therapy, experiencing three or more moderate/severe exacerbations per year and have BEC of ≥220 cells/μl, highlighting that clinical characteristics and BEC numerical thresholds vary between different drug classes (52). A complex interaction exists between clinical phenotype information, BEC, and mode of drug action to define COPD subgroups with increased likelihood of experiencing a beneficial response to immunomodulatory interventions. As the value of BEC as a biomarker for patients with COPD is now being recognized, incorporating eosinophil evaluation in future RCTs will allow further evaluation of BECs in different populations and with drugs that have different pharmacological actions.

## Conclusions

The evidence supports BEC as a biomarker that can be used in COPD RCTs of immunomodulatory treatments to predict treatment response. RCT analyses, both *post hoc* and prespecified, have demonstrated that drugs targeting eosinophils themselves and/or inflammation associated with eosinophils have a greater effect in individuals with greater BECs (23, 24, 36, 40, 52, 53, 80). Future RCTs with novel drugs targeting eosinophil-associated inflammation could use BEC to enrich the population with individuals who are more likely to benefit.

The interaction between clinical phenotype information and BEC means that a simple dichotomous approach to the future use of BECs in RCTs to predict treatment response is inappropriate (36, 80). Instead, careful consideration is needed regarding how the magnitude of treatment effect varies according to both the patients’ clinical characteristics and different BEC thresholds. This approach can be used to enrich future trial populations to potentially reduce drug development cost, trial failures, and trial patient numbers. For instance, the patient populations in recent ICS combination studies could have been reduced using BEC inclusion criteria. For drugs with other pharmacological mechanisms, the evidence already indicates that BEC may be applied to include or exclude subgroups with higher eosinophil-associated inflammation (23, 24, 52). Recent evidence that lower BECs are associated with increased risks of chronic bacterial infection and pneumonia indicates the potential of this biomarker to identify a subgroup for which strategies to target bacterial infection are required (22).

Precision medicine is the integration of clinical and biological information to optimize the benefit-versus-risk ratio for drug treatment. The Global Initiative for Chronic Obstructive Lung Disease (GOLD) recommendations include the use of BECs in clinical practice as a biomarker in conjunction with exacerbation risk for optimizing decisions regarding ICS use (9). These GOLD 2019 recommendations align with the concept of precision medicine. Furthermore, the complexity of the BEC–ICS response relationship is reflected in GOLD recommendations to use more than one threshold in clinical practice rather than a simple dichotomization of the population (9). The use of different thresholds is influenced by different degrees of exacerbation risk and whether the patient is already receiving one or two long-acting bronchodilators as different treatment alternatives exist for these scenarios. BECs provide an estimate of the probability of ICS benefit in these varied situations. Similarly, BECs can be used to identify subgroups with an increased probability of benefit in future RCTs of novel drugs that target eosinophil-associated inflammation. Biomarkers such as BEC should be applied to and validated in these future RCTs to facilitate precision medicine and improve the probability of successful drug development.

## Supplementary Material

Supplements

Author disclosures
