# Supplementary material for: Blood Eosinophil Counts in Clinical Trials for Chronic Obstructive Pulmonary Disease
Source: Am J Respir Crit Care Med. 2020 Sep 1;202(5):660–71. doi: 10.1164/rccm.201912-2384PP (PMC7462391; doi:10.1164/rccm.201912-2384PP)
Supplement: Supplements [file rccm.201912-2384PP.html]

Blood Eosinophil Counts in Clinical Trials for Chronic Obstructive Pulmonary Disease | American Journal of Respiratory and Critical Care Medicine

- disclosures.pdf (380 KB)
